# Affinity hierarchies and amphiphilic proteins underlie the co-assembly of nucleolar and heterochromatin condensates

**DOI:** 10.21203/rs.3.rs-3385692/v1

**Published:** 2023-09-29

**Authors:** Srivarsha Rajshekar, Omar Adame-Arana, Gaurav Bajpai, Kyle Lin, Serafin Colmenares, Samuel Safran, Gary H Karpen

**Affiliations:** 1 Department of Molecular and Cell Biology, University of California Berkeley, Berkeley, USA; 2 Department of Chemical and Biological Physics, Weizmann Institute of Science, Rehovot, Israel; 3 Max Planck Institute for the Physics of Complex Systems, Dresden, Germany; 4 Division of Biological Sciences and the Environment, Lawrence Berkeley National Laboratory, Berkeley, USA

## Abstract

Nucleoli are surrounded by Pericentromeric Heterochromatin (PCH), reflecting a close spatial association between the two largest biomolecular condensates in eukaryotic nuclei. This nuclear organizational feature is highly conserved and is disrupted in diseased states like senescence, however, the mechanisms driving PCH-nucleolar association are unclear. High-resolution live imaging during early Drosophila development revealed a highly dynamic process in which PCH and nucleolar formation is coordinated and interdependent. When nucleolus assembly was eliminated by deleting the ribosomal RNA genes (rDNA), PCH showed increased compaction and subsequent reorganization to a shell-like structure. In addition, in embryos lacking rDNA, some nucleolar proteins were redistributed into new bodies or ‘neocondensates,’ including enrichment in the core of the PCH shell. These observations, combined with physical modeling and simulations, suggested that nucleolar-PCH associations are mediated by a hierarchy of affinities between PCH, nucleoli, and ‘amphiphilic’ protein(s) that interact with both nucleolar and PCH components. This result was validated by demonstrating that the depletion of one candidate amphiphile, the nucleolar protein Pitchoune, significantly reduced PCH-nucleolar associations. Together, these results unveil a dynamic program for establishing nucleolar-PCH associations during animal development, demonstrate that nucleoli are required for normal PCH organization, and identify Pitchoune as an amphiphilic molecular link that promotes PCH-nucleolar associations. Finally, we propose that disrupting affinity hierarchies between interacting condensates can liberate molecules to form neocondensates or other aberrant structures that could contribute to cellular disease phenotypes.

## Introduction

The eukaryotic nucleus is organized into different membrane-less compartments known as biomolecular condensates that assemble via multivalent interactions between their constituent molecules. Prominent examples include the nucleolus, nuclear speckles, and heterochromatin, essential for cellular viability and function. An individual condensate can concentrate several distinct macromolecules in cells, including structured and intrinsically disordered proteins and nucleic acids (Lyon et al. 2021; Shin & Brangwynne 2017). Condensates with the same components that nucleate at different cellular locations can coarsen by fusions into larger clusters, whereas condensates with distinct compositions do not fuse (Ditlev et al. 2018). Nevertheless, in the crowded environment of the nucleus, distinct condensates can display close, conserved associations to form higher-order complex structures (Fare et al. 2021). Although many studies have examined the formation and function of individual condensates, we know relatively little about the interplay between distinct interacting condensates *in vivo* with respect to how they form and impact each other’s structure and function.

Two prominent nuclear condensates that are proximally located in a highly conserved manner are the Nucleolus and Pericentromeric Heterochromatin (PCH) (Bizhanova & Kaufman 2021; Padeken & Heun 2014). The nucleolus is a multi-layered biomolecular condensate that plays an essential role in the assembly of ribosomal subunits and forms by liquid-liquid phase separation of its constituent molecules (Brangwynne et al. 2011; Feric et al. 2016; Lafontaine et al. 2020). RNA-Polymerase I (RNA-Pol-I) transcribes tandemly-repeated ribosomal RNA genes (rDNA) to form the innermost layer (Fibrillar Center or FC), rRNA is processed by proteins such as Fibrillarin to generate the Dense Fibrillar Compartment (DFC), and ribosomal proteins assemble with processed rRNA to form ribosomes in the outermost layer, the Granular Compartment (GC) (Feric et al. 2016; Thiry & Lafontaine 2005). The different subcompartments recruit specific factors involved in rRNA transcription, processing, and ribosome assembly in a sequential manner to facilitate the vectorial expulsion of ribosomes through and out of the nucleolus (King et al. 2022; Riback et al. 2023). In most eukaryotic nuclei, the nucleolus is surrounded by PCH, a compact chromatin compartment that assembles on megabases of pericentromeric repeats, including tandemly repeated satellite DNA and transposable elements. While the nucleolus is the most transcriptionally active site in the nucleus, PCH is associated with transcriptional silencing and has essential roles in nuclear architecture and genome stability (Janssen et al. 2018). PCH is distinct from the inactive B compartment as defined by chromosome conformation capture experiments since Hi-C analyses typically exclude repetitive PCH sequences (Lieberman-Aiden et al. 2009). Under the microscope, PCH can be visualized as chromatin regions enriched for the AT-rich DNA dye DAPI, histone modifications di- and tri-methylation of histone H3 (H3K9me2/3), and the cognate epigenetic reader Heterochromatin Protein 1 (HP1) (Politz et al. 2013). HP1 is a multivalent protein with structured and disordered domains (Eissenberg & Elgin 2000) that undergoes phase separation *in vitro* and preferentially partitions DNA and nucleosomes (Keenen et al. 2020; Larson et al. 2017), while *in vivo,* it forms a liquid-like condensate nucleated by H3K9me2/3 enriched chromatin (Strom et al. 2017; Tortora et al. 2023; Wang et al. 2019).

A simple explanation for why PCH is organized close to the nucleolus is that tandem arrays of rDNA genes are positioned within pericentromeric satellite repeats on a subset of chromosomes (Potapova & Gerton 2019). However, proximity mapping nucleolus-associated domains (NADs) and IF-FISH revealed that PCH sequences from chromosomes without rDNA genes also associate with the nucleolus (Németh et al. 2010; Van Koningsbruggen et al. 2010). Thus, cis-proximity to rDNA is not necessary for PCH-nucleolar associations, and how PCH from all chromosomes becomes positioned around the nucleolus is unknown. Loss of the major nucleolar component nucleophosmin (NPM1) and its Drosophila homolog NLP in human and fly cell lines results in disorganization of nucleolus-associated heterochromatin (Holmberg Olausson et al. 2014; Padeken et al. 2013). Conversely, loss of major heterochromatin factors like Su(var)3–9 and HP1 impaired rDNA and nucleolar integrity (Ballmer et al. 2023; Peng & Karpen 2007), suggesting structural or functional relationships between the two domains. The proximal positioning of rDNA to PCH sequences is likely functionally relevant since not all rDNA genes are expressed, and a subset is silenced by heterochromatin formation (Srivastava et al. 2016). PCH repeats are dissociated from the nucleolus in senescent cells, suggesting that PCH-nucleolar association is linked to cellular health (Dillinger et al. 2017). Thus, the association of PCH and nucleoli is a prominent and conserved feature of 3D nuclear organization, but we lack mechanistic insight into how the two condensates co-assemble and affect each other’s structure and function.

This study uses live imaging and genetic tools in Drosophila embryos and tissues to reveal highly dynamic behaviors that characterize the *de novo* assembly of PCH around the nucleolus. During the initial phases of formation, PCH and nucleolar compartments are spatially separated. After independent growth and fusions, PCH first assembles at the nuclear edge and progressively reorganizes around the nucleolus as the embryo develops. Mutational analyses that perturb heterochromatin disrupt nucleolar shape and morphology. Reciprocally, embryos that lack rDNA and, thus, a functional nucleolus, show dramatic reorganization of PCH characterized by increased compaction followed by reorganization to a shell-like structure. *In vivo* experiments and physical modeling revealed that a hierarchy of interaction strengths between PCH, nucleoli, and ‘amphiphilic’ protein(s) able to interact with both nucleolar and PCH components generate the layered organization of nucleoli and PCH. This model led to identifying Pitchoune, a DEAD-box RNA-Helicase protein, as a molecular linker responsible for PCH-nucleolar associations. Finally, these results suggest that perturbed condensate associations and affinity hierarchies can generate abnormal cellular phenotypes in disease states.

## Results

### *De novo* establishment of PCH-nucleolar associations during early Drosophila embryogenesis is dynamic

**Figure 1:**
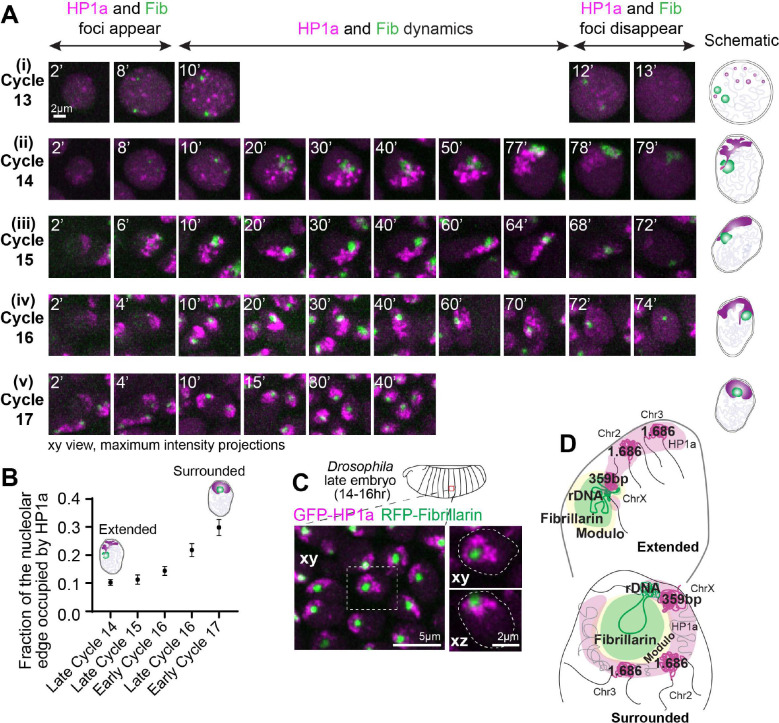
PCH reorganizes from the nuclear to the nucleolar edge during early Drosophila development. (**A**) Maximum intensity projections (xy view) of individual live nuclei of GFP-HP1a (magenta) and RFP-Fibrillarin (green) localization in Cycles 13–17 of Drosophila embryogenesis. The numbers on the top left corner of each image indicate time (in minutes) after mitotic exit. (**B**) Quantification of HP1a occupancy at the nucleolar edge during specific developmental cycles. “Early Cycle” refers to nuclei between 15–30 mins into the specified interphase, “Late Cycle” refers to nuclei between 50–70 mins. Data points = the mean, error bars = 95% confidence intervals around the mean. n>50 nuclei (from 5 embryos). at each time point. (**C**) Representative maximum intensity projections of PCH (GFP-HP1a, magenta) and nucleoli (RFP-Fibrillarin, green) in live epidermal nuclei in a late Drosophila embryo (Stage 16, ~14–16hr). The nucleus within the white dashed box is magnified on the right and presented in xy and xz views. White dashed lines indicate the nuclear boundary. (**D**) Schematic depicting the organization of the nucleolus and PCH in nuclei in early (top) and late (bottom) Drosophila embryos. 359bp and 1.686 repeats are major pericentromeric sequences on Chr X and Chr 2 & 3 respectively.

Although PCH and nucleolar assembly dynamics have been studied independently in Drosophila embryos (Falahati et al. 2016; Strom et al. 2017), they have not been analyzed simultaneously during *de novo* establishment. To determine how PCH is assembled de novo around the nucleolus, we performed high-resolution time-lapse imaging of early Drosophila embryos expressing RFP-Fib and GFP-HP1a transgenes as markers for the nucleolus and PCH, respectively. Drosophila embryos undergo 14 synchronized, syncytial nuclear divisions (S and M phases only) before cellularizing at blastoderm (cycle 14) and thereafter undergoing cell divisions (Farrell & O’Farrell 2014). Major chromatin features, including H3K9 methylation, are progressively established during the syncytial cycles (Ahmad & Henikoff 2022; Seller et al. 2019). PCH condensates first emerge in cycle 11 (Strom et al. 2017), while nucleoli first emerge in cycle 13, seeded by transcribing rRNA (Falahati et al. 2016), making cycle 13 the earliest time PCH and nucleolar condensates appear in the same nuclei. Upon entry in cycle 13 interphase, HP1a and Fibrillarin proteins are initially diffuse throughout the nucleus, then within ~8 mins, each becomes enriched in multiple, distinct foci (Figure 1A(i) and **Supplementary Movie 1**). Small PCH and nucleolar condensates remain separated throughout cycle 13, likely because growth is limited by the short interphase (~15 mins) before dissolution in mitosis.

**Extended Figure 1:**
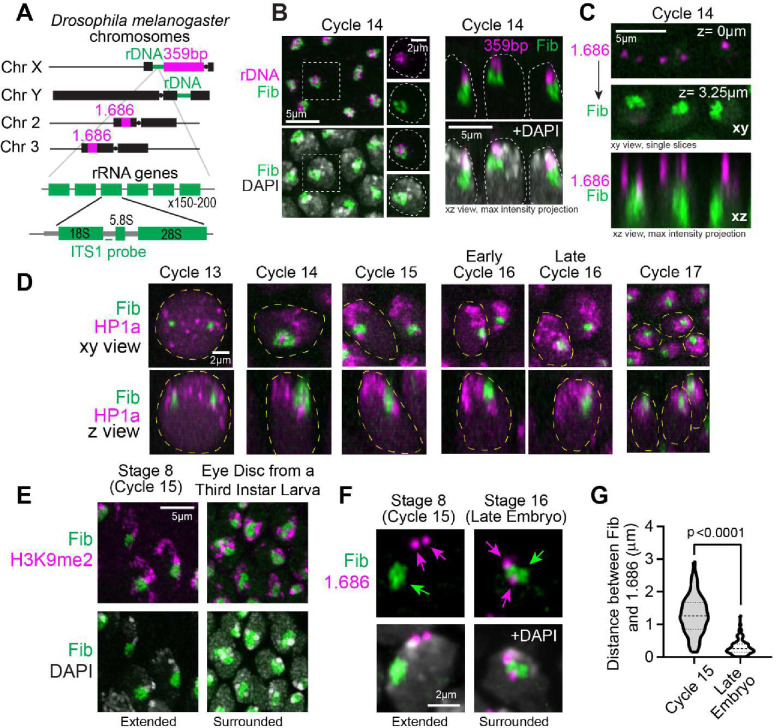
PCH reorganizes from the nuclear to the nucleolar edge during early Drosophila development. (**A**) Schematic of the locations of pericentromeric satellite repeats (359bp and 1.686) and rDNA repeats in Drosophila melanogaster chromosomes. Schematic of ribosomal DNA (rDNA) arrays in *Drosophila melanogaster* showing the position of the ITS-1 rDNA probe. (**B**) Combined immuno-FISH of Drosophila nuclei in Cycle 14 stained for Fibrillarin (green), DAPI (gray), (Left) ITS-1 rDNA (magenta), (Right) 359bp satellite DNA (magenta) on Chr X. Dashed white lines represent the edge of the nucleus determined by DAPI staining. (**C**) Combined immuno-FISH of Drosophila nuclei in Cycle 14 stained for Fibrillarin (green) and 1.686 satellite DNA (magenta) on Chr 2 and 3. (**D**) Representative stills of nuclei showing the conformation of the Fibrillarin and HP1a in progressive cycles 13–17. Nuclei marked with a dashed yellow boundary in the xy view are shown in their xz view below. (**E**) Immunofluorescence of H3K9me2 (magenta), Fibrillarin (green) and DAPI (gray) in nuclei from a Stage 8 (~Cycle 15) and eye disc from a third instar larva. (**F**) Combined immuno-FISH of 1.686 (magenta arrows), Fibrillarin (green arrow) and DAPI (grey) in a nucleus from epidermal cells of Stage 8 (~Cycle 15) and Stage 16 (Late) Drosophila embryos. (**G**) Distance between the centers of geometry of 1.686 and Fibrillarin in Stage 8 (~Cycle 15) and Stage 16 (Late) in Drosophila nuclei. n>60 loci (from 3 embryos) at each developmental stage.

Cycle 14 begins like cycle 13 in that HP1a and Fibrillarin foci emerge soon after mitotic exit and are initially separated. However, PCH and nucleolar condensates grow larger and undergo liquid-like self-fusions due to the extended length of cycle 14 interphase (~90 mins) (Falahati et al. 2016; Strom et al. 2017; Tortora et al. 2023) (Figure 1A(ii) and **Supplementary Movie 2**). By the end of cycle 14, a small section of the HP1a domain is associated with Fibrillarin, corresponding to the rDNA locus on the sex chromosomes (Extended Figure 1A–B). The majority of the HP1a domain, including chromosomes 2 and 3, is distributed apically and juxtaposed to the nuclear periphery (Figure 1A(ii) and Extended Figure 1C). This ‘extended’ conformation represents a previously undescribed intermediate during the establishment of PCH-nucleolar associations.

Continued live and fixed imaging of HP1a and Fibrillarin in post-blastoderm, asynchronous cell divisions revealed how the extended PCH configuration dynamically transitions into the canonical surrounded form observed in later developmental stages. As for cycles 13 and 14, in each post-blastoderm interphase (cycle 15 onwards), HP1a and Fibrillarin proteins are initially diffuse, then nucleate into separate foci that grow and fuse to form the extended intermediate. The PCH remains in the extended configuration and predominantly associates with the nuclear periphery through the rest of cycle 15 (Figure 1A(iii) and **Supplementary Movie 3**) but transitions between the extended and surrounded configurations during cycle 16 (Figure 1A(iv) and **Supplementary Movie 4**). Finally, stable formation of the surrounded organization is observed ~15 min into cycle 17 interphase (Figure 1A(v) and **Supplementary Movie 5**), after PCH condensates quickly transition through the extended configuration. Further analyses substantiate these observed live PCH-nucleolar dynamics. First, the fraction of the nucleolar edge occupied by HP1a increases between cycles 14 and 17 (Figure 1B and representative stills in Extended Figure D). Second, H3K9me2 displays the extended conformation relative to Fibrillarin in fixed Stage 8/cycle 15 embryos and localizes to the nucleolar edge later in development (Extended Figure 1E). Third, immuno-FISH revealed that satellite repeats 1.686 on Chr 2 and 3 are located farther from the nucleolus in cycle 15 (Stage 8; mean=1.28*μ*m) and display significantly more proximal localization in Stage 16 (mean=0.31*μ*m) (Extended Figure 1F–G).

**Extended Figure 2:**
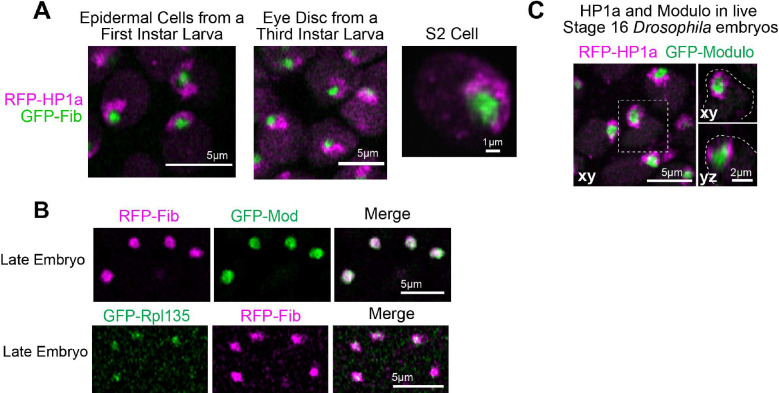
The nucleolus is surrounded by PCH in Drosophila nuclei. (**A**) Representative stills of nuclei from first instar larval epidermal cells (left), third instar larval eye disc (center), and S2 cell culture (right). Magenta, RFP-HP1a. Green, GFP-Fibrillarin. (**B**) Representative images of nucleoli showing the distribution of GFP-Modulo (green) and RFP-Fib (magenta) (top), and GFP-Rpl135 (green) and RFP-Fib (magenta) (bottom) in a live late (Stage 16) embryo. (**C**) Representative maximum intensity projections of PCH (RFP-HP1a, magenta) and nucleoli (GFP-Modulo, green) in live epidermal nuclei from a late Drosophila embryo (Stage 16, ~14–16hr). The nucleus within the white dashed box is magnified on the right and presented in xy and xz view. White dashed lines indicate the nuclear boundary.

Live imaging in late embryos (~12–16 hr, Embryonic Stage 14–16), larval tissues, and cultured S2 cells expressing fluorescently tagged transgenes for HP1a revealed that the canonical surrounded conformation persists in later developmental stages (Figure 1C and Extended Figure 2A). *Drosophila* nucleoli are also multi-layered as in other eukaryotes; the GC is marked by Modulo (the fly ortholog for Nucleolin), Fibrillarin marks the DFC, and Rpl-135, a subunit of RNA-Pol-I localizes within the Fibrillarin compartment (Extended Figure 2B). HP1a/PCH organizes around the outermost nucleolar layer (GC) in late Drosophila embryos (Extended Figure 2C). Together, we conclude that PCH-nucleolar associations are highly dynamic during each interphase; PCH and nucleolar proteins are diffuse upon mitotic exit, then both condensates undergo independent nucleation, growth, and fusions, and display cycle-specific spatiotemporal differences while transitioning from the extended (nuclear periphery) to the surrounded (nucleolar periphery) configurations (summarized in Figure 1D, schematic).

### Reduced levels of heterochromatin result in decondensed rDNA and enlarged nucleoli

**Extended Figure 3:**
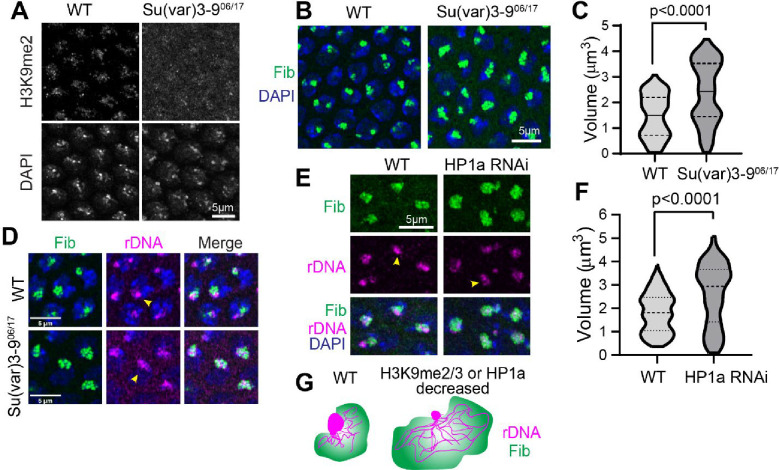
The nucleolus is enlarged and rDNA is decondensed when H3K9 methylation or HP1a is reduced. (**A**) Maximum intensity z projections of WT and Su(var)3–9^06/17^ nuclei immunostained with H3K9me2 (green) and co-stained with DAPI (blue) at nuclear cycle 14. (**B**) Maximum intensity z projections of nuclei immunostained with Fibrillarin (green) and costained with DAPI (blue) in WT and Su(var)3–9^06/17^ embryos at Cycle 14. (**C**) Quantification of the volume of Fibrillarin in WT and Su(var)3–9^06/17^ embryos at Cycle 14. (**D**) Combined immuno FISH (maximum intensity z projections) of Fibrillarin (green), rDNA (magenta) and DAPI (blue) in WT and Su(var)3–9^06/17^ embryos at Cycle 14. (**E**) Maximum intensity z projections of nuclei from wildtype and HP1a RNAi embryos at Cycle 14. Fibrillarin (green), rDNA (red), DAPI (blue). (**F**) Volume of Fibrillarin in WT and HP1a RNAi in embryos at Cycle 14. (**G**) Schematic of the observed phenotypes depicting decondensation of rDNA and nucleolar enlargement when H3K9me2/3 or HP1a is decreased.

Is the organization of PCH and nucleolar condensates interdependent? We investigated whether PCH is required for proper nucleolus formation by removing one of the major H3K9 methyltransferase genes. Su(var)3–9^06/17^ embryos (Schotta et al. 2002) generated from Su(var)3–9^06/17^ mothers are depleted for H3K9me2 in cycle 14 (Extended Figure 3A) and display a 1.5-fold increase in nucleolar volume compared to wild-type controls (Extended Figure 3B–C). In addition, combined immuno-FISH analysis revealed that the high-intensity rDNA block located at the nucleolar edge in wild-type is reduced in Su(var)3–9^06/17^ embryos, with corresponding increases in the amount of dispersed rDNA within the nucleolus (Extended Figure 3D). Similarly, nucleolar volume increased in cycle 14 after maternal knockdown of HP1a using RNAi (Extended Figure 3E–F). We conclude that PCH constrains nucleolar size and limits rDNA distribution within the nucleolus (summarized in Extended Figure 3G). This is consistent with previous studies that show HP1/H3K9me2/3 is enriched at silent rDNA in flies and mammalian cells (Fefelova et al. 2022; Santoro et al. 2002) and that HP1 maintains nucleolar structure by excluding repeats from the nucleolus (Ballmer et al. 2023). Further research is required to determine if the absence of H3K9me2/3 and HP1 alters nucleolar morphology by de-silencing rRNA transcription versus a requirement for physical association with PCH condensates.

### Embryos lacking nucleoli display abnormal PCH organization and neocondensate formation

**Figure 2:**
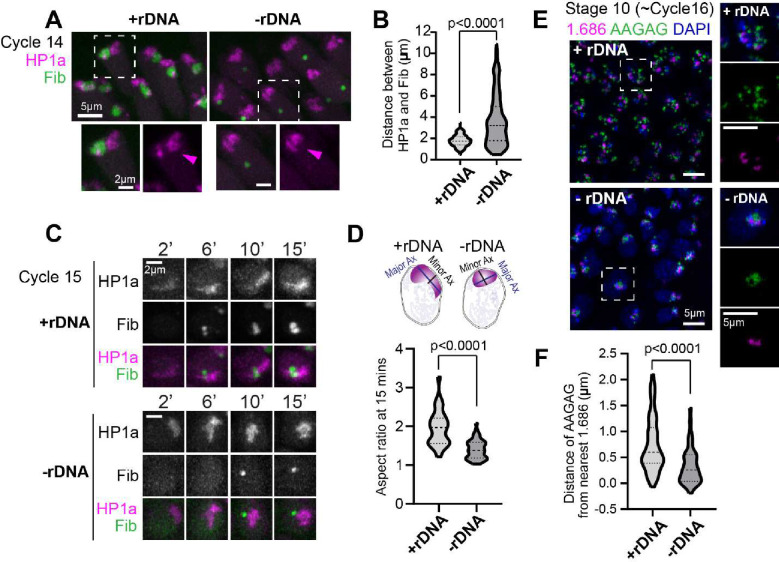
Increased PCH compaction in embryos lacking rDNA. (**A**) Maximum intensity projections of live nuclei from wildtype embryos (+rDNA) and mutant embryos lacking rDNA (−rDNA) at Cycle 14, showing RFP-HP1a (magenta) and GFP-Fibrillarin (green). The dashed box highlights the nuclei enlarged in the panel below. (**B**) Distance between the centers of geometry of HP1a and Fibrillarin in +rDNA and −rDNA embryos. n>200 nuclei (from 5 embryos) for each genotype. (**C**) Maximum intensity projections from live imaging of nuclei from +rDNA and −rDNA embryos showing the reassembly of RFP-HP1a (magenta) and GFP-Fibrillarin (green) at the indicated minutes after the start of Cycle 15. (**D**) Quantification of the aspect ratio (major axis over minor axis) of HP1a segments 15 minutes after the start of Cycle 15 in +rDNA and −rDNA embryos. n>30 nuclei in each genotype. (**E**) Maximum intensity projections of FISH (Fluorescence In Situ Hybridization) of 1.686 (magenta) and AAGAG (green) in DAPI (blue)-stained nuclei in +rDNA and −rDNA Stage 10 embryos. The dashed boxes mark the nuclei enlarged on the right. (**F**) Quantification of the distance between AAGAG and its nearest 1.686 locus. n>40 pairs of loci in each genotype.

Next, we determined if nucleolar condensates impact PCH organization by imaging RFP-HP1a and GFP-Fib in embryos that completely lack rDNA repeats (designated hereafter as −rDNA) due to the presence of a rearranged X chromosome (C(1)DX/0, Valencia et al., 1949). No functional nucleoli are formed in −rDNA embryos (Falahati et al. 2016), but they develop into late-stage embryos due to maternal deposition of ribosomes. While no nucleolus is present, Fibrillarin forms spherical structures (as previously reported by Falahati et al. 2016), which are located at a significantly longer distance from the HP1a/PCH condensate compared to +rDNA controls (Figure 2A–B; +rDNA mean=1.78*μ*m, −rDNA mean=3.69*μ*m, p<0.0001). This result suggests that Fibrillarin and HP1a proteins do not interact directly and, thus, are unlikely to mediate the canonical surrounded organization. Instead, we propose that when factors responsible for nucleolus nucleation (rDNA/rRNA) are not present, Fibrillarin self-associations (Berry et al. 2015) and/or secondary affinities with other structures or molecules are sufficient to cause the formation of new enrichments not found in wild-type cells (hereafter termed ‘neocondensates’).

Most importantly, PCH condensate morphology is significantly perturbed in embryos lacking rDNA. The “extended” HP1a conformation normally observed in cycles 14 and 15 is replaced by a more compact, rounded structure at the apical end of −rDNA nuclei (Figure 2A, C). The aspect ratio (major axis/minor axis) of the HP1a domain is significantly reduced (Figure 2D; +rDNA mean=1.96, −rDNA mean=1.40, p<0.0001), and the distance between pericentromeric repeats 1.686 (Chr 2 and 3) and AAGAG (all chromosomes) is decreased in −rDNA nuclei compared to +rDNA controls (Figure 2E–F; +rDNA mean=0.74*μ*m, −rDNA mean=0.32*μ*m, p<0.0001)). We conclude that nucleoli are required to avoid hyper-compaction of PCH condensates in early Drosophila embryos (before the end of cycle 14).

**Extended Figure 4:**
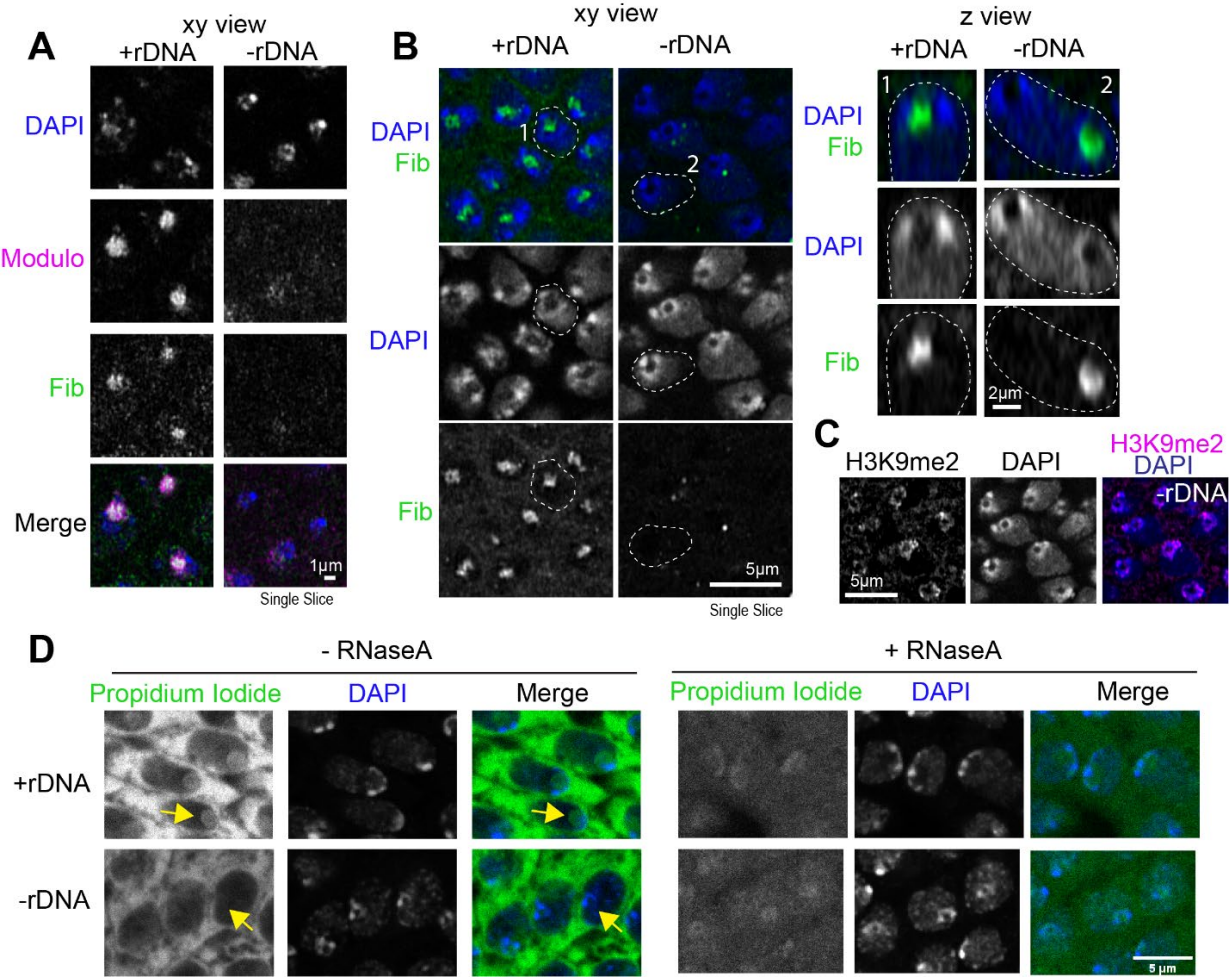
The HP1a-void in −rDNA embryos does not stain for DAPI, Fibrillarin, Modulo or Propidium Iodide (RNA). (**A**) Representative images of fixed nuclei from wildtype late embryos and mutant embryos lacking rDNA stained for Modulo (magenta), Fibrillarin (green) and DAPI (blue). (**B**) Left: Representative images of fixed nuclei from Stage 14–16 (late) wildtype embryos and mutant embryos lacking rDNA showing Fibrillarin (green) and DAPI (blue). Right: Nuclei marked with white dashed outlines on the left are shown in the z plane. (**C**) H3K9me2 immunofluorescence (magenta) and DAPI (blue) staining in −rDNA nuclei in Stage 14–16 (late) Drosophila embryos. (**D**) Representative images of fixed nuclei from Stage 14–16 (late) wildtype and mutant embryos lacking rDNA stained with Propidium Iodide (green) and DAPI (blue) without (left) and with (right) RNaseA. The yellow arrow points to the RNA staining in the nucleolus in +rDNA and lack of propodium iodide staining in −rDNA nuclei.

**Figure 3:**
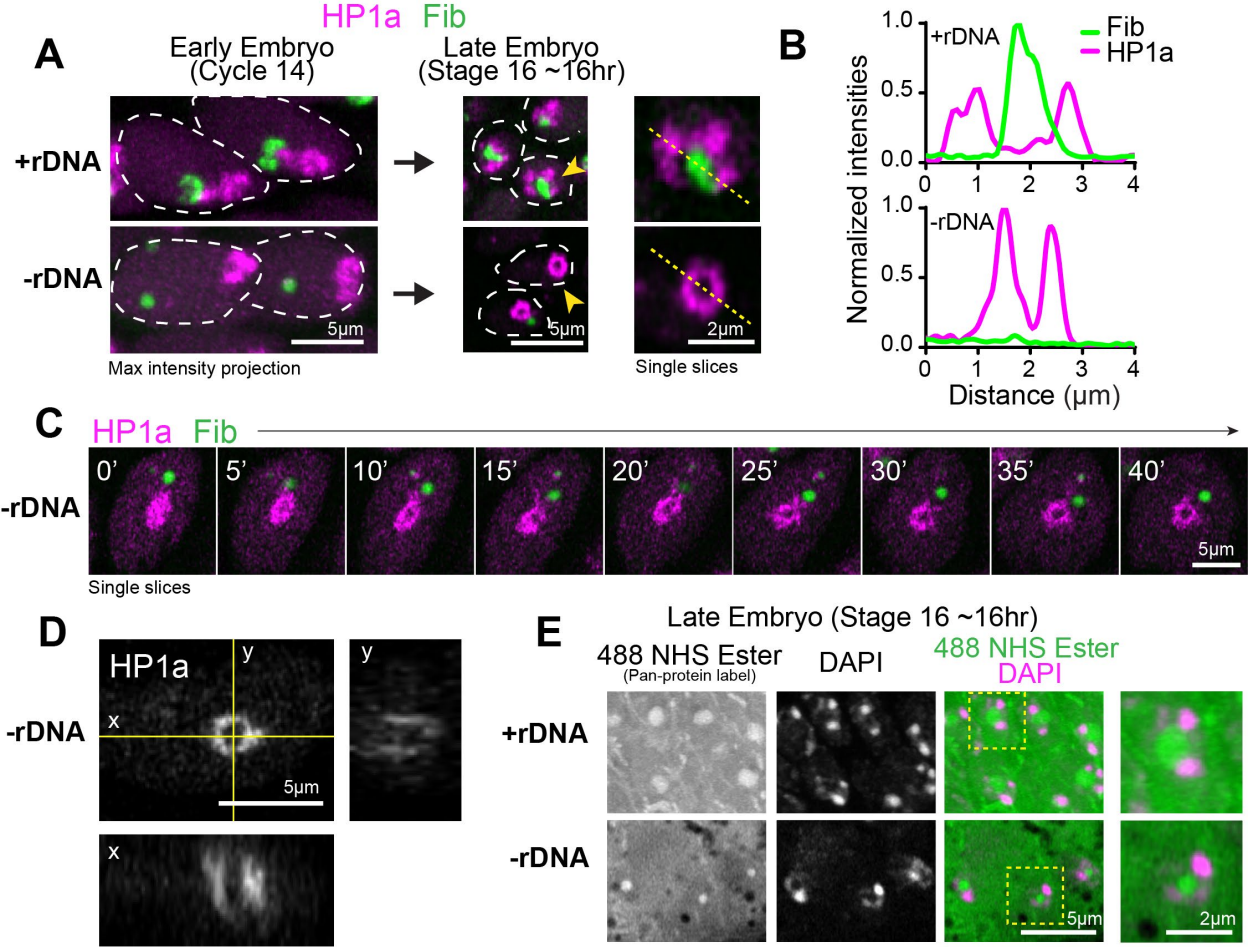
A protein-filled core reshapes the PCH condensate to a sphere-like structure in −rDNA developing embryos. (**A**) Representative stills of live +rDNA and −rDNA nuclei at Cycle 14 and in Stage 16 (late stage) embryos showing RFP-HP1a (magenta) and GFP-Fibrillarin (green). Nuclei marked with the yellow arrowhead are enlarged on the right. (**B**) Line scans of the intensity along the dashed yellow line in the optical slices shown in (A). (**C**) Time-lapse stills (single slices) of a −rDNA amnioserosa nucleus with RFP-HP1a (magenta) and GFP-Fibrillarin (green). (**D**) Orthogonal views (single slices) of RFP-HP1a in a −rDNA live amnioserosa nucleus. The yellow lines indicate the location of the orthogonal slices. (**E**) Late embryos (Stage 16) with the +rDNA or −rDNA genotype was co-stained with a pan-protein label, 488 NHS Ester (green) and DAPI (magenta). Nuclei marked with the black dashed box are enlarged on the right.

Unexpectedly, in late-stage −rDNA Drosophila embryos, the PCH morphed from a hyper-compacted cluster to a hollow sphere-like structure, surrounding a core devoid of HP1a (hereafter ‘PCH void’) (Figure 3A–B). To visualize this transformation at increased spatial resolution, we performed live imaging in the amnioserosa, whose large nuclei form a monolayer on the dorsal surface of the embryo during gastrulation (Lacy & Hutson 2016). Live imaging of the amnioserosa nuclei in −rDNA Drosophila embryos revealed the stepwise transformation of the compacted PCH to the formation of a progressively larger PCH void (Figure 3C–D and **Supplementary Movie 6**). In addition to the absence of HP1a, the PCH void lacked the major nucleolar proteins Fibrillarin (Figure 3A–B) and Modulo (Extended Figure 4A), which formed neocondensates or dispersed in the nuclear space, respectively. The PCH void did not contain DNA (DAPI staining), histones marked with H3K9me2 (IF), or any significant accumulation of RNAs (Propidium Iodide staining) (Extended Figures 4B–D). However, treatment with 488 NHS Ester, a pan-protein label (M’Saad & Bewersdorf 2020), revealed that the PCH void was enriched for unknown proteins (Figure 3E). We conclude that PCH initially displays an atypical, hyper-compacted morphology in embryos lacking rDNA and nucleoli. Further, the PCH morphs into an abnormal sphere-like structure whose central core (the PCH void) lacks HP1a, nucleolar proteins, chromatin, and RNA but is filled with unknown protein(s) that may represent another neocondensate. Importantly, these results suggest that nucleoli are required for the normal distribution and organization of PCH condensates in nuclear space and that disrupting interactions within or between condensates can create new nuclear structures.

### Coarse-grained modeling recapitulates *in vivo* phenotypes and highlights a potential role for amphiphilic proteins in mediating PCH-nucleolar organization

**Figure 4:**
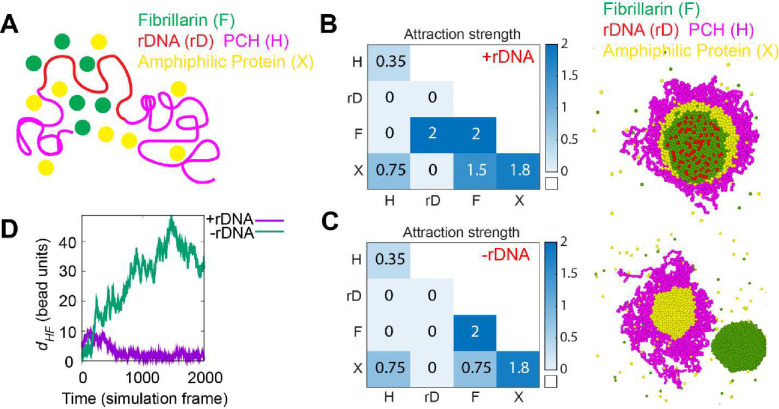
A hierarchy of interaction strengths between PCH, nucleoli and ‘amphiphilic’ protein(s) recapitulates in vivo +rDNA and −rDNA phenotypes. (**A**) Course-grained modelling of nucleolar-PCH assembly with four minimal components: PCH (H) as a self-interacting polymer (magenta) rDNA (rD) as polymeric block embedded within PCH (red), Fibrillarin (F) as a representative of a self-associating nucleolar protein (green), and an ‘amphiphilic’ protein (X) that has an affinity for both PCH and nucleolar components (yellow). (**B and C**) The matrix indicates the strength of interaction (blue gradient, units: k_B_T) between Fibrillarin (F), rDNA (rD), PCH (H) and an unknown amphiphilic protein (X), with simulated outcomes for +rDNA and −rDNA conditions on the right. (**D**) Distance (d_HF_) between the centers of mass of PCH (H) and Fibrillarin (F) clusters in +rDNA and −rDNA simulations.

We used coarse-grained molecular dynamics simulations and physical modeling to identify the molecular interactions and biophysical parameters that could mediate nucleolar-PCH associations in wild-type embryos, as well as PCH disorganization in the absence of rDNA. Based on our experimental observations and previous studies, the model was constrained by four main features. First, some nucleolar proteins like Fibrillarin have an intrinsic tendency to form condensates (Figure 2A) (Falahati et al. 2016; Feric et al. 2016). Second, PCH forms a condensate that associates with the nucleolus (Figure 1) (Strom et al. 2017). Third, PCH and Fibrillarin have no direct associations (Figure 2A). Fourth, unknown proteins accumulate in the PCH void in −rDNA embryos (Figure 3E). We thus modeled four minimal components (see details in Materials and Methods) (Figure 4A): (i) PCH (H) as a long, self-interacting polymer, (ii) rDNA (rD) as a polymeric block within the chromatin fiber that experiences good solvent conditions and is flanked by two PCH blocks, (iii) Fibrillarin (F), representative of a self-associating nucleolar protein, and (iv) an ‘amphiphilic’ protein (X), defined here as having shared affinities for both PCH and nucleolar components (etymology: Greek for ‘likes both’). Similar ‘amphiphilic’ proteins have previously been synthesized and demonstrated to form multiphasic condensates in vitro (Kelley et al. 2021).

**Extended Figure 5:**
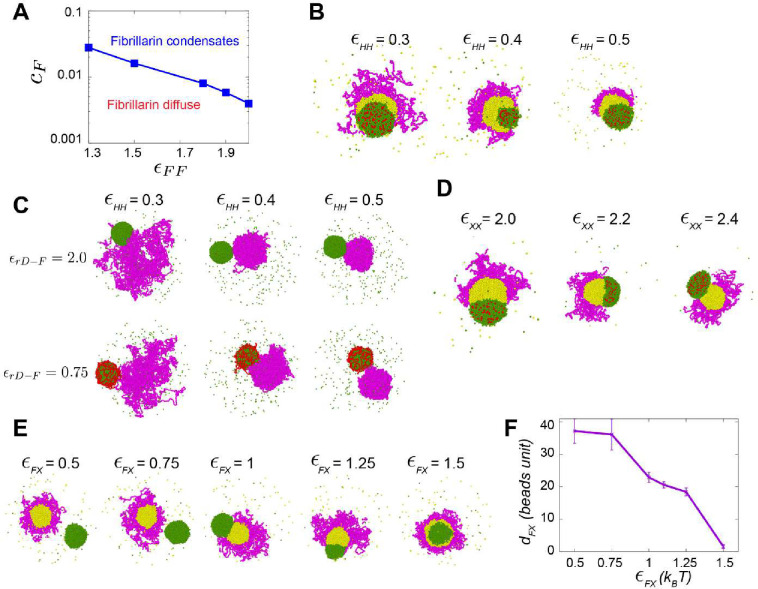
Coarse-grained model for the assembly of the nucleolus and PCH. (**A**) The phase diagram illustrates the minimum attraction strength required to condense Fibrillarin for different concentrations. (**B**) Simulation endpoint snapshots depict the outcomes of varying the attraction strengths between beads of the PCH (H) polymer chain $\left(\epsilon_{HH}\right)$. (**C**) Simulation snapshots depict varying attraction strengths between rDNA-Fibrillarin (top to bottom) and beads of the PCH polymer chain (left to right). (**D**) Simulation endpoint snapshots depict the outcomes of varying X-X attraction strengths $\left(\epsilon_{XX}\right)$. (**E**) Simulation endpoint snapshots depict the outcomes of varying attraction strengths between Fibrillarin and protein $X\;\left(\epsilon_{FX}\right)$ in the −rDNA condition. (**F**) The average distance $\left(d_{FX}\right)$ between Fibrillarin and protein X condensates from their center of mass is measured for different attraction strengths. Error Bars represent SD.

The theory of three-phase wetting of simple liquids demonstrates that a balance between the interfacial tensions of the different liquids determines the spatial configurations of the phases (Torza & Mason 1969). Thus, we propose that to form the layered PCH-nucleolar organization, the self-associations of Fibrillarin must be stronger than those of the amphiphilic protein, which in turn must be stronger than the self-interactions of PCH, since interfacial tension is proportional to the relative interaction strengths. These considerations led us to define a hierarchy of interaction strengths (rD-F=F-F>X-X>F-X>X-H>H-H) as a foundation for simulations using the parameters listed in the 4×4 interaction matrix (Figure 4B). Importantly, simulating this affinity hierarchy recapitulated the canonical PCH-nucleolar organization observed in +rDNA animals *in vivo (*Figure 4B and **Supplementary Movie 7**). Various scenarios incompatible with the experimental observations of the layered organization of the nucleolus and PCH arise by changing the hierarchy of self-interaction strengths in the simulations (Extended Figure 5). The detailed rationale for the choice of parameters is described in the Methods section.

Setting the rDNA-Fibrillarin interactions to zero to model the consequences of eliminating the rDNA resulted in the formation of the protein X neocondensate within the PCH void and the spatially separated Fibrillarin neocondensate (Figure 4C–D), as observed in vivo (Figures 2 and 3). These phenotypes are observed in the simulations whenever the protein X – PCH interaction strength is greater than or equal to the X – Fibrillarin interactions (i.e., X-H≥F-X) (Extended Figure 5E–F). In the model, the nucleation of the new phase of amphiphilic protein X occurs within the PCH to reduce the cost of creating an interface of protein X facing the solvent. Instead, being surrounded by the PCH can reduce this interfacial energy, as expected from a wetting picture of simple liquids (Torza & Mason 1969). In sum, simply eliminating the rDNA from this minimal model, and thus the ability to recruit nucleolar components such as Fibrillarin, recapitulates both the PCH void filled with X protein and Fibrillarin neocondensate phenotypes observed in −rDNA nuclei *in vivo*.

We conclude that incorporating affinity hierarchies and an amphiphilic protein X into a minimal model recapitulates the observed *in vivo* organization of PCH and nucleolar condensates in both +rDNA and −rDNA conditions (Figure 4B, C). Further, the experimental and simulation results indicate that the same amphiphilic protein(s) could mediate the surrounded configuration in wild-type and accumulation in the PCH void in the −rDNA condition. This led us to predict that protein X should be a self-associating, outer nucleolar/granular compartment protein with an affinity for PCH components, such as HP1a.

### The DEAD-box RNA Helicase Pitchoune is a granular compartment protein that forms a neocondensate within PCH in −rDNA embryos

**Figure 5:**
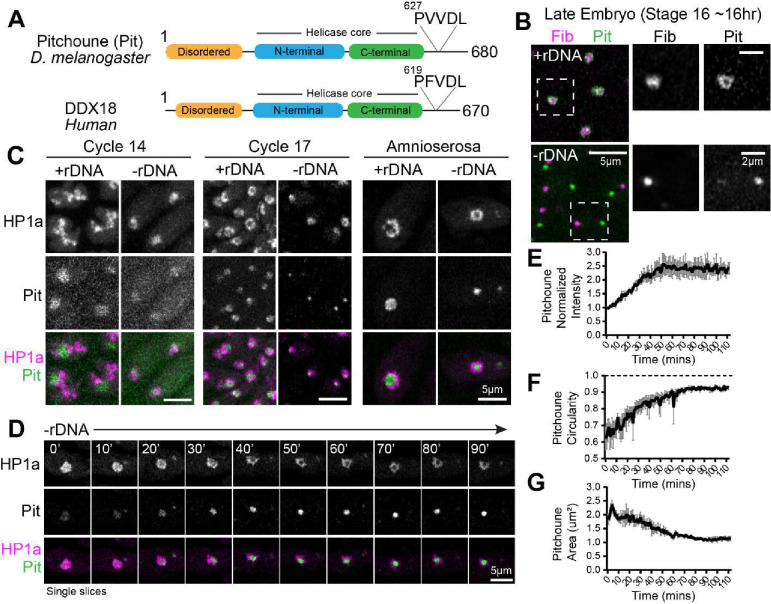
The DEAD-Box RNA Helicase Pitchoune is enriched in the PCH void in −rDNA embryos. (**A**) Protein subdomains including a conserved PXVXL HP1-interaction motif in Pitchoune (Pit) and its human ortholog DDX18. (**B**) Representative images of epidermal nuclei from late-stage Drosophila embryos with +rDNA and −rDNA genetic backgrounds with RFP-Fibrillarin (magenta) and Pitchoune-GFP (green). Nuclei marked with the white dashed box are enlarged on the right. (**C**) Maximum intensity projections of Cycle 14, Cycle 17 and amnioserosa nuclei from +rDNA and −rDNA embryos showing RFP-HP1a (magenta) and Pitchoune-GFP (green). (**D**) Time-lapse stills (single slices) of a −rDNA amnioserosa nuclei with RFP-HP1a (magenta) and Pitchoune-GFP (green). (**E**) Mean intensity, (**F**) Circularity, and (**G**) Area of projections of Pitchoune in −rDNA embryos. Mean of 35–60 nuclei at each time point in n= 3 embryos. Error Bars: SEM.

The physical modeling results predicted that amphiphilic proteins interacting with both PCH and the nucleolus would be enriched in the PCH void in −rDNA embryos. A candidate for such a protein emerged in Falahati & Wieschaus (2017) who reported that the DEAD-Box RNA Helicase nucleolar component Pitchoune (Pit) (Zaffran et al. 1998) mislocalized in cycle 14 −rDNA embryos to the apical part of the nucleus. We reasoned that it could be co-localizing with the hyper-compacted PCH observed in the absence of nucleoli, suggesting an affinity for PCH (Figure 2A). Moreover, Pitchoune and its human ortholog DDX18 have an N-terminal disordered domain that could mediate self-association and/or nucleolar interactions, a helicase core, and a consensus PxVxL motif found in many HP1a binding partners (Figure 5A) (Thiru et al. 2004). To test the hypothesis that Pitchoune acts as the predicted amphiphilic protein X *in vivo*, we performed live imaging in Drosophila embryos expressing RFP-Fibrillarin and Pitchoune-GFP transgenes (Falahati & Wieschaus 2017). In +rDNA, Pitchoune localizes to the nucleolus with enrichment at the edge of Fibrillarin, suggesting that Pitchoune is enriched in the granular compartment, thus juxtaposed with the surrounding PCH (Figure 5B). However, in −rDNA embryos, Pitchoune and Fibrillarin do not associate and form separate, high-intensity spherical bodies (Figure 5B), suggesting that Pitchoune does not interact with Fibrillarin in the absence of rDNA and rRNA.

To investigate whether Pitchoune is enriched in the PCH void in −rDNA embryos, we performed live imaging in +rDNA and −rDNA embryos co-expressing Pit-GFP and RFP-HP1a transgenes. In cycle 14 +rDNA nuclei, Pitchoune localizes to the nucleolus while HP1a is in the extended form away from the nucleolus. In contrast, in −rDNA nuclei, a faint Pitchoune signal co-localizes with HP1a (Figure 5C). As the embryo develops, HP1a appears in the canonical form surrounding Pitchoune in +rDNA embryos. However, in −rDNA embryos, high-intensity Pitchoune puncta appear within each compacted HP1a condensate in cycle 17 epidermal nuclei and amnioserosa nuclei (Figure 5C). This revealed that the Pitchoune puncta filling the PCH void are first visible in cycle 17 in −rDNA embryos, the same developmental stage where the stable surrounded configuration is established in +rDNA embryos.

Time-lapse imaging of amnioserosa nuclei in −rDNA embryos revealed that the intensity of Pitchoune within HP1a is initially low and increases ~2.5-fold over an hour (Figure 5D–E and **Supplementary Movie 8**). Furthermore, the circularity of Pitchoune increases and approaches 1 (in projections) (Figure 5F), and the size of Pitchoune decreases over time (Figure 5G), suggesting that Pitchoune demixes from PCH to form a sphere, thereby minimizing its surface area due to its interfacial tension. Together, these data suggest that the nucleation and growth of Pitchoune within PCH creates a neocondensate whose emergence coincides with the formation of the PCH-void in −rDNA embryos. Notably, the predictions of the modeling results were validated *in vivo* and led to the discovery of Pitchoune as an example of a new class of amphiphilic proteins that interact with the nucleolus and PCH.

### Knockdown of Pitchoune decreases PCH-nucleolar associations

**Figure 6:**
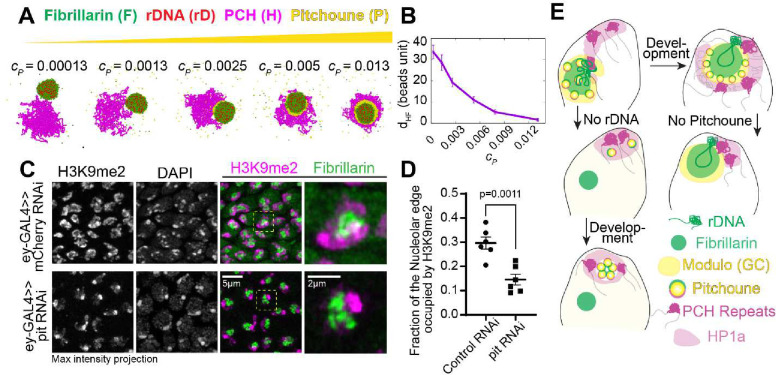
Knockdown of Pitchoune decreases PCH association with the nucleolus. (**A**) Simulation endpoint snapshots demonstrate how decreasing the concentration (c_P_) of Pitchoune (yellow) reduces the association of PCH (H) (magenta) around the Fibrillarin condensate (F) (green). (**B**) The average distance (d_HF_) between the PCH (H) and Fibrillarin (F) clusters from their center of mass is measured at different Pitchoune concentrations (c_P_). Error Bars represent standard deviation (SD). (**C**) Immunofluorescence of H3K9me2 (magenta), Fibrillarin (green) and DAPI in nuclei from dissected eye-discs in third instar larvae from UAS-Pitchoune RNAi and UAS-mCherry RNAi (control) driven by the eyeless-GAL4 driver. (**D**) Quantification of the fraction of the nucleolar edge occupied by H3K9me2 in 3D. Each data point indicates the mean value from one animal. n=6 animals with over 500 nuclei in total for each experimental group. Error bars indicate SEM. (**E**) Schematic of the model showing the impact of −rDNA and Pitchoune knockdown on the organization of PCH and nucleoli.

While the *in vivo* experiments and simulations aligned to reveal that Pitchoune has affinities for both PCH and the nucleolus, we next asked whether Pitchoune is required for PCH-nucleolar associations. We first investigated this by decreasing the concentration of the amphiphilic protein in our physical model while keeping all other parameters the same as in Figure 4B. The simulations revealed a progressive clustering of PCH away from the nucleolus as Pitchoune levels decreased (Figure 6A–B). However, unlike −rDNA (Figure 4C), PCH maintained a restricted attachment to Fibrillarin in these conditions since the rDNA polymer is embedded between PCH blocks. These results led us to hypothesize that Pitchoune could act as a linker protein for stabilizing PCH-nucleolar associations in the ‘surrounded’ conformation.

**Extended Figure 6:**
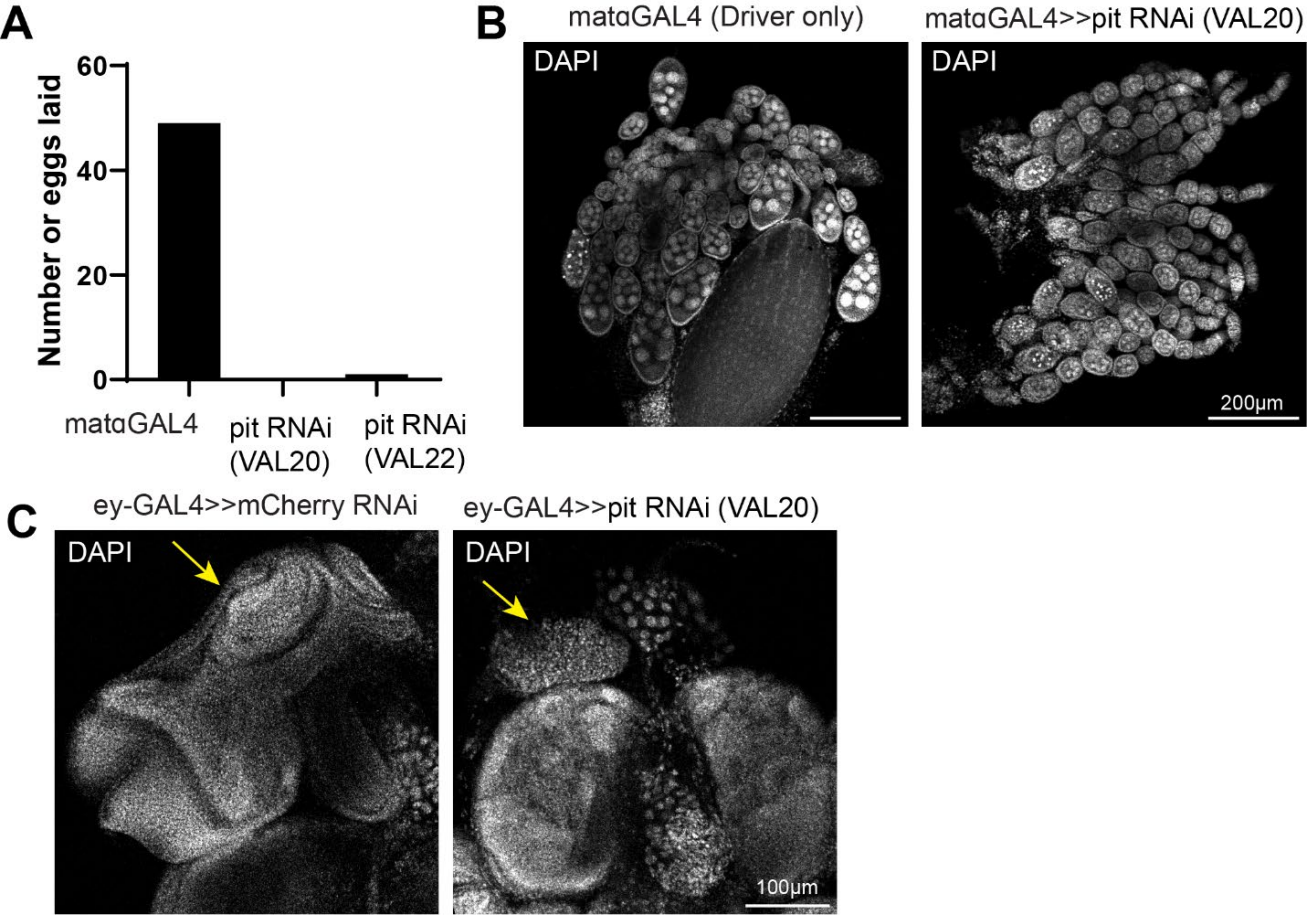
Knockdown of Pitchoune in the female germline and eye-discs prevents their normal development. (**A**) Number of eggs laid on an apple juice plate after a 3hr collection in mataGAL4 (driver only) and mataGAL4 driving UAS-Pitchoune RNAi (VAL20 and VAL22 lines). (**B**) Representative images of dissected ovaries in control (mataGAL4, driver only) and after Pitchoune knockdown stained with DAPI. (**C**) Representative images of dissected eye antennal discs (yellow arrow) in control (ey-GAL4>mCherry RNAi) and after Pitchoune knockdown (ey-GAL4>pit RNAi, VAL20) stained with DAPI.

To test this hypothesis *in vivo*, we depleted Pitchoune protein levels using RNAi in Drosophila tissues. When we knocked down Pitchoune in the female germline using a maternal GAL4 driver, no eggs were laid (Extended Figure 6A), and ovary development was halted (Extended Figure 6B), precluding further assessment of the effects of Pitchoune knockdown in early embryos. Therefore, we used eyeless-GAL4 to knock down Pitchoune in eye-antennal discs, representing a tissue where nuclei form the canonical surrounded PCH-nucleolar conformation. In third-instar larvae, upon Pitchoune knockdown, the normal development of the eye-antennal discs is severely perturbed (Extended Figure 6C), consistent with defects in cell proliferation (Zaffran et al. 1998). Crucially, the PCH-nucleolar organization recapitulated the physical prediction for the depletion of an amphiphilic molecule linking the PCH and nucleolus. Control samples showed H3K9me2 organized around the nucleolus. However, upon Pitchoune knockdown, both DAPI and H3K9me2 patterns changed, becoming more compact and rounded while still attached to Fibrillarin (Figure 6C). We observed a 50% reduction in the fraction of the nucleolar edge in 3D occupied by H3K9me3 after Pitchoune knockdown (control RNAi mean=0.296, pit RNAi mean=0.146, p-value=0.0011) (Figure 6D). Together, these results reveal a new mechanism for the association of two distinct biomolecular condensates and demonstrate a previously unappreciated role for the RNA Helicase Pitchoune in promoting PCH organization around the nucleolus.

## Discussion

This study presents four main findings about the organization of pericentromeric heterochromatin (PCH) and nucleolar condensates in the developing Drosophila embryo. Firstly, we observed that PCH-nucleolar associations are highly dynamic during initial establishment in early embryos, characterized by cycle-specific spatio-temporal differences in transitions between the extended (nuclear periphery) and surrounded (nucleolar periphery) configurations (Figure 1). Secondly, we have established a structural relationship between these two condensates, discovering that while PCH plays a role in restricting nucleolar size, the nucleolus facilitates the distribution of PCH components and repeats within the nuclear space (Figure 2). Thirdly, we have shown that a hierarchy of interaction strengths between nucleolar and PCH components could regulate their layered organization. Notably, the model predicted previously unrecognized protein(s) with ‘amphiphilic’ properties, which exhibit binding affinities to both the nucleolus and PCH (Figure 3–4). Lastly, we identified Pitchoune, a DEAD-box RNA-Helicase, as a potential amphiphilic protein that stabilizes PCH-nucleolar associations and demonstrated that Pitchoune removal reduces nucleolar-PCH interactions and disrupts the surrounded configuration (Figure 5–6). These findings provide mechanistic insights into the co-assembly dynamics and organization of PCH and nucleolar condensates, contributing to our understanding of a prominent conserved feature of nuclear architecture (Figure 6E).

The multiphasic nature of the nucleolus has been elegantly demonstrated, where multiple, immiscible layered phases form due to localized transcription and processing of rRNA, advective flow or vectorial transport, and layer-specific protein-protein and protein-rRNA binding (Feric et al. 2016; King et al. 2022; Lafontaine et al. 2020; Riback et al. 2023). Our study introduces a new mechanism for layering PCH around the nucleolus in which some nucleolar components with affinities for PCH can stabilize physical associations between the nucleolus and PCH. Further, we identified the DEAD-Box RNA Helicase, Pitchoune, as such an amphiphilic protein that localizes to the granular compartment of the nucleolus, contains a C-terminal PXVXL HP1a binding motif, and importantly, is required for PCH-nucleolar ‘surrounded’ configurations. Pitchoune’s affinity for PCH is further supported by observing initial mixing with HP1a in cycle 14 and, later, the consistent formation of new spherical structures (neocondensates) within PCH in nuclei lacking rDNA. It is important to emphasize that Pitchoune is not restricted only to the nucleolar-PCH interface but is localized through the outer nucleolar layer, reminiscent of the behavior of synthetic amphiphilic proteins at higher concentrations that form 3D multiphasic protein condensates instead of monolayer films (Kelley et al. 2021). Together, we posit that PCH-nucleolar condensate juxtaposition arises from the balance between Pitchoune nucleolar associations and weaker interactions with HP1, and that Pitchoune’s amphiphilic nature stabilizes physical associations between immiscible condensates by simple minimization of the interfacial energy. Further studies are required to determine if this phenomenon of amphiphilic protein-mediated interactions between distinct condensates is generalizable.

The physical model accounted for the formation of the PCH void in −rDNA embryos through self-association of the amphiphilic protein, combined with lower affinity binding to PCH. Although Pitchoune self-association has not been demonstrated, oligomerization of orthologous RNA-dependent DEAD-Box ATPases (DDXs) does promote the formation of RNA-rich condensates (Overwijn & Hondele, 2023). Interestingly, Pitchoune neocondensates and the PCH void emerge in –rDNA embryos at the same timepoint (cycle 17) when the canonical ‘surrounded’ PCH-nucleolar conformation appears in +rDNA embryos. We thus hypothesize that Pitchoune’s ability to mediate PCH-nucleolar associations is altered at this developmental stage, perhaps via increased self-association, abundance (beyond saturation), or increased affinity for HP1. Such changes could elevate the energy cost of creating an interface with the nucleoplasm/solvent and promote associations with PCH condensates by minimizing this interfacial energy. Finally, we note the structural similarity between the PCH voids observed in −rDNA Drosophila embryos and Nucleolus Precursor Bodies (NPBs) observed in mammalian oocytes and early embryos (Fulka & Aoki 2016). Since they appear before the initiation of rRNA synthesis, we speculate that NPBs are formed by DDX-rich neocondensates, as observed for Pitchoune in the absence of rDNA and nucleoli.

Our research unveils the process of *de novo* co-assembly of the nucleolus and PCH at high spatial and temporal resolution in the naïve chromatin landscape of a developing Drosophila embryo. PCH and nucleolar condensates are nucleated independently and are initially spatially separated. As they grow and coarsen (self-fuse), one end of PCH is tethered to the nucleolus via rDNA while the rest spreads along the nuclear edge to form an ‘extended’ intermediate. Subsequently, PCH from all chromosomes transitions to the canonical ‘surrounded’ configuration around the nucleolus. Associations between uniquely mapped heterochromatin and the lamina (nuclear periphery) have been well characterized (van Steensel & Belmont, 2017), and recent studies have demonstrated overlaps between Nucleolus-associated domains (NADs) and Lamina-associated domains (LADs) (Bersaglieri et al., 2022; Bizhanova et al., 2020; Vertii et al., 2019). Our work uniquely captures the dynamics of the transitions of PCH between the nuclear and nucleolar periphery during embryonic development. Although the precise mechanisms of these dynamics are yet to be elucidated, we speculate that changes in chemical and biophysical properties, such as varying nucleolar and PCH condensate volumes or material properties, increasing H3K9me2/3 levels, rRNA expression levels, or post-translational modifications of molecular factors like Pitchoune, might alter binding affinities, influencing the balance of PCH’s association with the nucleolus versus the lamina.

The nucleolus has been recognized as a nuclear hub that attracts heterochromatin (Padeken & Heun 2014; Quinodoz et al. 2018). Our study expands on this knowledge by revealing that the nucleolus is required to distribute PCH condensates and repeats in the 3D nuclear space since removing the nucleolus causes increased PCH compaction, potentially allowing PCH self-attractions to dominate (Figure 3). The distribution of PCH repeats around the nucleolus is likely functionally important, as the increased compaction could restrict critical nuclear processes like PCH transcription, repair, or replication. Reciprocally, genetic depletion experiments in the early Drosophila embryo revealed that the loss of H3K9me2/3 and HP1a causes rDNA decondensation and nucleolar enlargement (Supplementary Figure 3). This result could indicate that PCH condensates directly impact nucleolar structure and function. However, we favor the view that nucleolar enlargement reflects the role of heterochromatin in silencing the transcription of subsets of rDNA repeats and the partitioning of silent rDNA to nucleolus-associated heterochromatin (Németh & Längst 2011; Pontvianne et al. 2013; Santoro et al. 2002; Van Koningsbruggen et al. 2010). This does not preclude the possibility that the surrounding PCH has independent roles in nucleolar functions, such as impact on ribosome export. Together, we propose a model where PCH and nucleoli connected via a chromatin polymer (rDNA) regulate each other’s spatial organization; the nucleolus distributes the PCH in the nucleus while the PCH constrains nucleolar size by limiting rDNA transcription and may impact other nucleolar functions.

Finally, we predict that during aging and senescence, and perhaps other disease states, the balance of interactions between PCH and nucleoli is perturbed to cause cellular phenotypes such as enlarged nucleoli (Buchwalter & Hetzer 2017), decreased HP1a and heterochromatin (Larson et al., 2012), and the dissociation of PCH repeats from the nucleolus (Dillinger et al. 2017). While these cellular aging phenotypes are correlated, our model predicts a causative relationship between PCH and nucleolar changes, which remains to be tested. Additionally, our findings demonstrate how disrupting condensate nucleation sites or interaction hierarchies can form new, abnormal nuclear structures or ‘neocondensates’ through inherent self-associations or secondary interactions with other molecules. Removing rDNA causes Modulo to become broadly dispersed in the nuclear volume, while Fibrillarin forms a spherical body separated from the PCH, and a Pitchoune neocondensate transforms the compacted PCH into a shell. Since the cell contains several multi-component condensates, our results may have broader relevance since perturbing one set of interactions could give rise to new condensates/aggregates that cause defective cellular phenotypes or behaviors. Understanding these outcomes will also be relevant in cellular stress responses when new condensates are often formed or the composition of existing condensates changes (Alberti & Hyman 2021).

## MATERIALS AND METHODS

**Table:** 

Reagent or Resource	Source	Identifier
Antibodies		
Rabbit anti-Fibrillarin	Abcam	ab5821
Mouse anti-H3K9me2	Abcam	ab8898
Mouse anti-Modulo	Gift from Mellone Lab (gift of Jacques Pradel, Chin-Chi Chen et al., 2012)	
Goat-anti-Mouse, Alexa Fluor 488	Invitrogen	A-11001
Goat-anti-Mouse, Alexa Fluor 568	Invitrogen	A-11004
Goat-anti-Rabbit, Alexa Fluor 488	Invitrogen	A-11034
Donkey-anti-Rabbit, Alexa Fluor 568	Invitrogen	A-10042
**Bacterial Strains**		
DH5α Competent Cells	This study	N/A
**Chemicals**		
DAPI	Sigma	D9542–10MG
Triton-X	Sigma	T8787
Commercial Bleach	Clorox	N/A
Formaldehyde Solution	Sigma	F8775
Halocarbon Oil 27	Sigma	H8773
Gas-permeable Lumox Film	Sarstedt	Catalog # 94.6077.317
Heptane	Sigma	246654
Methanol	VWR (EMD)	EM-MX0485–9
Bovine Serum Albumin	VWR	0332
VECTASHIELD^®^ Antifade Mounting	Vector	H-1000–10
Medium	Laboratories	
Atto 488 NHS Ester	Sigma	41698
Propidium Iodide Solution	Invitrogen	P3566
**Critical Commercial Assays**		
FISH Tag^™^ DNA Multicolor Kit,	Thermo Fisher	F32951
Alexa Fluor^™^ dye combination		
pGEM T-Easy Cloning Kit	Promega	A1360
**Recombinant DNA**		
pGEM-rDNA	This study	N/A
**FISH Probes**		
359bp	This study	N/A
1.686	This study	N/A
AAGAG	This study	N/A
rDNA	This study	N/A
**Experimental Models and Subject Details**		
**Fly Genotype**	**Source**	
Oregon-R	BDSC # 5	
RFP-HP1a (2nd Chr)	Lipsick Lab (Wen et al., 2008)	
GFP-HP1a (3rd Chr)	Lipsick Lab (Wen et al., 2008)	
GFP-Fibrillarin	Weischaus Lab (Falahati and Wieschaus, 2017)	
RFP-Fibrillarin	Weischaus Lab (Falahati et al., 2016)	
GFP-Modulo	Weischaus Lab (Falahati and Wieschaus, 2017)	
Pitchoune-GFP	Weischaus Lab (Falahati and Wieschaus, 2017)	
GFP-Rpl135	Weischaus Lab (Blythe et al., 2015)	
Su(var)3–906/ TM3Ser	Reuter Lab (Tschiersch et al., 1994)	
Su(var)3–917/ TM3Ser	Reuter Lab (Tschiersch et al., 1994)	
Mat alpha GAL4	BDSC # 7063	
HP1a RNAi	BDSC # 33400	
FM6/C(1)DX, y[*] f[1]	BDSC # 784	
C(1)RM/C(1;Y)6,y[1]w[1]f[1]/0	BDSC # 9460	
Eyeless-GAL4	BDSC # 5534	
Pitchoune RNAi VAL20	BDSC # 80368	
Pitchoune RNAi VAL22	BDSC # 43984	
**Primers**		
rDNA FISH: 5’-ACGGTTGTTTCGCAAAAGTT-3’ and 5’-TGTTGCGAAATGTCTTAGTTTCA-3’		
**Software and Algorithms**		
ImageJ (Fiji)	NIH	
ZEN Zeiss	Zeiss	https://www.zeiss.com/microscopy/int/products/microscope-software/zen.html
Arivis Vision 4D	Arivis	https://www.arivis.com/
GraphPad Prism	GraphPad	https://www.graphpad.com/

### Method Details

#### Drosophila Stocks and Genetics

All crosses were maintained at 25° C. To visualize the dynamics of HP1a and nucleolar assembly, live embryos from stocks containing *RFP-HP1a; GFP-Fib*, *RFP-Fib; GFP-HP1a* and *RFP-HP1a; GFP-Mod* were imaged. Embryos lacking rDNA were obtained as described in Falahati () by crossing C(1)DX/Y; *RFP-HP1a; GFP-Fib* or C(1)DX/Y; *RFP-HP1a; Pit-GFP* or C(1)DX/Y; *RFP-Fib; Pit-GFP* virgins to C(1;Y)6,y[1]w[1]f[1]/0 males. 1/4^th^ of the resulting embryos from this cross lack rDNA, and −rDNA embryos were selected based on the presence of Fibrillarin neocondensates in live and fixed embryos and DAPI morphology in fixed embryos.

To knockdown HP1a in Drosophila embryos, we crossed matα-GAL4 (BDSC #7063) females to HP1a RNAi males (BDSC #33400) to generate an F1 generation with HP1a depleted in the female germlines. F1 siblings were crossed to collect embryos depleted for HP1a, most of which exhibit severe developmental defects (Park et al. 2019). We selected embryos that reached nuclear cycle 14 for analyses of nucleolar morphology. The study of the effects of HP1a knockdown on the nucleolus was also limited to cycle 14 since most embryos were arrested by this stage. To analyze nucleolar phenotypes in Su(var)3–9^null^ embryos, we crossed Su(var)3–9^06^ /TM3Ser virgins to Su(var)3–9^17^ /TM3Ser males. After selecting Su(var)3–9^06/17^ adults in the F1 generation, we intercrossed these siblings to collect Su(var)3–9^06/17^ maternal null embryos. However, H3K9me2/3 levels partially recover by the gastrula stage (cycle 15) (through the action of other H3K9me2/3 methyltransferases like Eggless (Seller et al. 2019)), which limited this analysis to cycle 14. To knockdown Pitchoune in eye discs, eyeless-GAL4 virgin females were crossed with Pitchoune RNAi VAL20 males, and eye discs were dissected from F1 third instar larvae.

#### Live Imaging of Drosophila Embryos

To collect Drosophila embryos for live imaging, males and females of the desired genotype were added to a plastic cage covered with apple juice agar plates and left for at least 3 days at 25°C. On the day of imaging, a fresh plate was added to the cage, and embryos were collected for two hours. After removing the plate, embryos were coated with Halocarbon oil 27 (Sigma) for staging using a dissection scope with transillumination, then stage-selected embryos were placed on a 2 in × 2 in paper towel square and dechorionated in 50% bleach for 1 min. Bleach was wicked off with a Kimwipe after 1 min, the square was washed with a small amount of distilled water, and excess water was wicked off. Dechorionated embryos were secured onto a semipermeable membrane (Lumox film, Starstedt) on a membrane slide holder using Heptane Glue. These embryos were mounted in Halocarbon oil 27 (Sigma) between the membrane and a coverslip. Live imaging was performed on a Zeiss LSM880 Airy Scan microscope (Airy Fast mode) with a 63X oil immersion objective at room temperature.

#### Immunostaining

Embryos were collected on apple juice-agar plates and aged till the appropriate stage, dechorionated in 50% bleach, fixed in 1:1 heptane:4% formaldehyde in 1XPBS for 25 mins, devitellinized in a 1:1 mixture of methanol:heptane, and stored at −20°C in methanol. Embryos were rehydrated by washing in 1xPBS+0.2% Triton (PBT), blocked for ~1hr with 2% BSA in PBT, then incubated with the primary antibody at 4°C overnight. Following washes with PBT, embryos were incubated with the appropriate secondary antibody at room temperature for 2 hrs, stained in DAPI, and mounted onto a slide using VectaShield (Vector Laboratories) mounting medium. All primary antibodies were used at 1:250 dilution and secondaries at 1:1000. Imaging was performed on a Zeiss LSM880 Airy Scan microscope (Airy Fast mode) with 63X oil immersion objective at room temperature.

#### DNA Fluorescent in situ hybridization (FISH) and combined Immuno-FISH

##### Probe Labelling

A probe for ITS-1 rDNA was prepared by amplifying an ~800 bp fragment by PCR from genomic DNA using the primers 5’-ACGGTTGTTTCGCAAAAGTT-3’ and 5’-TGTTGCGAAATGTCTTAGTTTCA-3’ that was cloned into a pGEM-Teasy vector (Promega). Using this plasmid as a template, ITS-1 rDNA probe was labeled with Alexa 488, Alexa 555, or Alexa 648 using the FISH Tag^™^ DNA Multicolor Kit (ThermoFisher) following the manufacturer’s protocol. Locked nucleic acid (LNA) oligonucleotides (Integrated DNA technologies) conjugated with Cy5 or FAM were used as probes for 359bp, 1.686, and AAGAG satellite DNA repeats.

##### Hybridization

Embryos were collected on apple juice-agar plates and aged till the appropriate stage, dechorionated in 50% bleach, then fixed in 1:1 heptane: 4% formaldehyde in 1XPBS for 25 mins, devitellinized in a 1:1 mixture of methanol: heptane, and stored in −20°C. Embryos were washed in 2xSSC-T (2xSSC containing 0.1% Tween-20) with increasing formamide concentrations (20%, 40%, then 50%) for 15 min each. 100ng of DNA probes in 40 μl of hybridization solution (50% formamide, 3× SSCT, 10% dextran sulfate) was added, denatured together with the embryos at 95°C for 5 min and incubated overnight at 37°C. Following hybridization, embryos were washed twice in 2xSSCT for 30 mins at 37°C and thrice in PBT for 5 mins at room temperature. After completing washes, embryos were stained in DAPI and mounted onto a slide using VectaShield (Vector Laboratories) mounting medium.

##### Combined Immuno-FISH

For combined in situ detection of proteins and DNA sequences, immunofluorescence was performed first, embryos were post-fixed in 4% formaldehyde for 25 mins, then processed for FISH.

#### Pan-Protein Staining using 488 NHS Ester

Formaldehyde-fixed embryos were devitellinized in a 1:1 mixture of methanol: heptane and stored in methanol. Embryos were rehydrated by washing in 1xPBS+0.2% Triton (PBT). After washing off methanol, embryos were stained in the diluted 488 NHS ester fluorophore (1:50) from a 10mg/ml stock in 0.1% PBST for 6 h at 4 °C followed by washing in PBT three times for 30 mins each at room temperature. Embryos were stained in DAPI for ten minutes and mounted onto a slide using VectaShield (Vector Laboratories) mounting medium.

#### Propidium Iodide Staining

Formaldehyde-fixed and Heptane devitellinized embryos were rehydrated by washing in 1xPBS+0.2% Triton (PBT). Samples were equilibrated in 2X SSC. RNase-treated controls alone were incubated in 100 μg/mL DNase-free RNase in 2X SSC for 20 minutes at 37°C. After washing away the RNase with 2X SSC, embryos were incubated in 500nM of Propidium Iodide in 2X SSC for 10 mins at room temperature. Samples were rinsed in 2X SSC, stained with DAPI, and mounted on a slide with VectaShield (Vector Laboratories) mounting medium.

#### Modeling

To better understand the association of PCH with the nucleolus, we developed a physical model that simulates the interactions between different types of molecules found in these biomolecular condensates. In our physical model, we simulate four components of the nucleus: PCH (H) and ribosomal DNA (rD) as long polymers, and Fibrillarin (F) and an amphiphilic protein (X) as independent, single monomers, which we hereafter refer to as beads. Since the experiments focused on the PCH domain of Drosophila, our physical model only simulates this specific region of the genome (30% of the genome), not the entire genome. This allows us to study the dynamics of this particular region of the genome more accurately and efficiently. PCH is modeled as a semiflexible bead-spring polymer chain in which N beads are connected by N-1 harmonic springs. Each bead of the chain represents a cluster of PCH containing approximately 5 kilo base pairs of DNA, with a diameter of approximately 30 nm. The semi-flexibility of the chain is determined by its persistence length, which is taken to be 60 nm (2 beads) in accordance with previous studies that indicate chromatin has a persistence length between 50 to 100 nm (Wachsmuth et al. 2016). We represent rDNA as a self-avoiding chain that occupies approximately 20% of the middle domain of PCH. Fibrillarin and the amphiphilic protein X are modeled using single, diffusive beads where each protein has distinct interactions with the polymer and other proteins.

To simplify the model, we assume that the size of each protein bead is equal to the size of the heterochromatin bead, with both having a diameter *σ*. The non-bonding interactions between polymer-polymer, protein-protein, and polymer-protein are modeled using a standard Lennard-Jones (LJ) potential. The LJ potential is truncated at the distance 2.5 *σ*, meaning that the interaction between the beads is only non-zero if they are within 2.5 *σ* distance. At very short distances between the beads, the LJ potential is strongly repulsive (representing the excluded volume of two molecules). At intermediate spacings, the LJ potential is attractive, with a strength adjusted to model the different states of chromatin, such as its compaction or decondensation and the phase-separating tendency of the proteins. In our model, the polymer and the proteins are confined within a spherical boundary that represents the nucleus of the cell. This boundary mimics the effect of the nuclear envelope, which constrains the movement of these beads and affects their interactions with each other. In our study, we used the LAMMPS (Large-scale Atomic/Molecular Massively Parallel Simulator) package to simulate the behavior of our biomolecular system (Thompson et al. 2022). LAMMPS uses Brownian dynamics, which accounts for the viscous forces acting on the beads, and a stochastic force (Langevin thermostat) to ensure that the system of beads and solvent is maintained at a constant temperature (NVT ensemble). This allows us to model the interactions between polymer-polymer, polymer-protein, and protein-protein beads accurately and study the behavior of the system over time.

#### Rationale for the choice of parameters in the coarse-grained model

To analyze the experimental observations of phase separation in the nucleus, we study a minimal model with four crucial components: PCH (H), rDNA (rD), Fibrillarin (F), and an amphiphilic protein (X) that binds both nucleolar and PCH components. The parameters in the simulations include the number of molecules of each component $\alpha \;\left(N_{\alpha }\right)$, the strength of the attractions between two components (denoted by indices β and $\left.\gamma ,\epsilon_{\beta \gamma }\right)$, and the size of the confinement $\left(R_{c}\right.)$ The fraction of the nucleus that is hydrated (does not contain PCH or the other proteins) is obtained from the relative difference between the confinement volume and the volumes of PCH and the other proteins.

##### Bonding potential between monomers in the polymer made of PCH and rDNA:

Adjacent beads on the polymer chain are interconnected by harmonic springs using the potential function:

$$V_{s}=\sum_{i=1}^{N-1}k_{s}{\left(r_{i}-\sigma \right)}^{2}$$

Here, $r_{i}$ represents the distance between the i-th and (i+1)-th beads. The spring constant and equilibrium distance between neighboring beads are denoted as $k_{s}$ and σ respectively. In our simulations, the spring constant $k_{s}$ is set to $100k_{B}T/\sigma^{2}\;$ to ensure the presence of rigid bonds between adjacent beads of the polymer chain.

##### Attraction strength $(\epsilon_{\beta \gamma }):$

The Lennard-Jones potential is used to model the attraction between any two non-bonded beads:

$$V_{\beta \gamma }(r)=\{\begin{array}{cc} 4\epsilon_{\beta \gamma }\left[{\left(\frac{\sigma }{r}\right)}^{12}-{\left(\frac{\sigma }{r}\right)}^{6}\right] & \text{for}\;r\leq r_{c}\\ 0 & \text{for}\;r>r_{c} \end{array}$$

Here, the symbol $\epsilon_{\beta \gamma }$ represents the attraction strength between beads of type β and γ, where β,γ∈{H,rD,F,X} represent PCH, rDNA, Fibrillarin, and amphiphilic protein, respectively. For instance, $\epsilon_{FX}\;$ represents the attraction strength between Fibrillarin and amphiphilic protein beads. When dealing with attractive interactions between chromatin-chromatin and chromatin-protein beads, a distance cutoff of $r_{c}=2.5\sigma$ is used for the Lennard-Jones potential, beyond which the interaction is set to zero. To account for only excluded volume interactions (with no attractions) using the same potential, a cutoff of $r_{c}=2^{1/6}\sigma$ and $\epsilon =1k_{B}T$ are employed. This choice is made because the potential energy is at its minimum at that point, and the resulting force on a bead is zero. As there are four components in our model, there are a total of 10 combinations ((n(n+1)/2), where n is number of components) of attraction strength parameters between the different components.

##### Confinement size:

The size of the confinement is determined by defining the volume fraction of chromatin ϕ:

$$\phi =N_{G}\times \frac{\text{volume}\;\text{of}\;1\;\text{bead}\;}{\text{volume}\;\text{of}\;\text{confinement}\;}=N_{G}\times \frac{\left(\frac{4}{3}\pi \sigma^{3}\right)}{\left(\frac{4}{3}\pi R_{c}^{3}\right)}$$

Here, $N_{G}$ represents the total number of beads in the Drosophila genome, where each bead corresponds to 5 kilobase pairs (kbps) of DNA. The diameter of a spherical bead, denoted as σ, is taken to be 30 nm (see Bajpai & Safran, 2023 for further explanation). The total length of the diploid Drosophila genome is 360 Mbps, and $N_{G}$ can be calculated by dividing the Drosophila genome length by the amount of DNA represented by one bead (5 kbps). The volume fraction of chromatin $\left(\phi \right)$ is commonly reported as ~ 0.1 in existing literature (Qi & Zhang, 2021; Tripathi & Menon, 2019). Using the equation above, the calculated radius of the confinement $\left(R_{c}\right)\;$ is found to be 45σ.

##### Number of molecules in PCH and rDNA:

We model the PCH domain as a polymer chain composed of N=10,000 beads, which represents approximately 30% of the beads in the entire genome. Among these 10,000 beads, 20% are designated as rDNA, resulting in a total of 4,000 rDNA beads. We do not explicitly model the rest of the genome since the experiments show that the nucleolar components are localized near PCH.

##### Concentration of Fibrillarin and amphiphilic protein:

The concentration of Fibrillarin and amphiphilic protein is calculated as follows:

$$\text{concentration}\;\left(c_{\alpha }\right)=\frac{N_{\alpha }}{\text{volume}\;\text{of}\;\text{confinement}\;}$$

Here, the symbol α represents the type of protein, where $c_{\alpha }=c_{F}$ for the fibrillarin concentration and $c_{\alpha }=c_{X}$ for the amphiphilic protein concentration. After defining the parameters and obtaining the value for the confinement radius parameter from the assumed volume fraction of PCH, we proceed with an initial simulation, focusing on a single component, namely the Fibrillarin protein, within the confinement. During this simulation, we vary the concentration $\left(c_{F}\right)$ and the attraction strength $\left(\epsilon_{FF}\right)$ of Fibrillarin. Our experimental results demonstrate that Fibrillarin undergoes phase separation independently of the other components (rDNA or PCH) during cycle 14 (refer to Figure 2A). These initial-stage simulation results yield a phase diagram, which indicates that a minimum attraction strength of $1.3-2.0k_{B}T$ is required to condense fibrillarin particles within the concentration range of $c_{F}=0.0013-0.013$. The reported concentration of nucleolar particles is c=0.015 (Qi & Zhang, 2021). Consequently, we maintain a fixed concentration of Fibrillarin at $c_{F}=0.013$ when simulating all the other protein components (Extended Figure 5A). Since the concentration of amphiphilic protein is not determined from the experiments, we conducted multiple simulations, systematically varying the amphiphilic protein concentration $\left(c_{X}\right)$ within the range of 0.0013 − 0.13. The results of these simulations are discussed in Figure 6A.

##### Parameter range of $\epsilon_{HH}$:

To understand the behavior of each component separately (before including interactions between different components) in the next stage, we conducted simulations specifically focusing only on the PCH chain. The PCH chain represents a condensed chromatin region within the nucleus which implies self-attractive interactions of the beads representing the polymer. During these simulations, we varied the attraction strength between PCH beads $\left(\epsilon_{HH}\right)$ from 0 to $0.5k_{B}T$. Our results revealed that within the range of $\epsilon_{HH}=0.35-0.5k_{B}T$ (Extended Figure 5B), the PCH chain underwent collapse, resulting in a condensed conformation that is phase-separated from the aqueous component of the system (not simulated explicitly).

##### Parameter value of $\epsilon_{rD-F}$:

We next simulate the self-organization due to the interactions between the three components (where each component so far was considered alone): PCH, rDNA, and Fibrillarin. We set $\epsilon_{HH}=0.35k_{B}T$ and $\epsilon_{FF}=2k_{B}T$, incorporating only excluded-volume interactions (hard-core, repulsive interactions) between H-F, i.e., there is no direct attraction between PCH and Fibrillarin as implied by the experiments. By varying the attraction strength between rDNA and Fibrillarin $\left(\epsilon_{rD-F}\right)$, we made the following observations based on our simulation results: a weaker attraction (and $\epsilon_{rD-F}=0.75k_{B}T$) resulted in rDNA wrapping around the condensed Fibrillarin phase, while a stronger attraction (and $\epsilon_{rD-F}=2k_{B}T$) led to the condensation of rDNA within the Fibrillarin complex. This latter observation aligns with our experimental findings. Therefore, we select $\epsilon_{rD-F}=2k_{B}T$ as the parameter value in the subsequent simulations (Extended Figure 5C).

##### Parameter ranges of $\epsilon_{FF}$ and $\epsilon_{XX-}$:

Finally, we introduce the fourth component, an amphiphilic protein ‘X’, which we suggest may interact attractively with both PCH and Fibrillarin. Initially, we investigated the relative attraction strengths between Fibrillarin and protein X when considering the same concentration for both. We explore all possible combinations of $\epsilon_{FF}$ both greater than and less than $\epsilon_{XX}$. For $\epsilon_{XX}\geq \epsilon_{FF}$, we observe PCH surrounding the amphiphilic protein-rich phase, but just a partial wetting between the amphiphilic protein-rich phase and the Fibrillarin-rich phase (Extended Figure 5D). Only when $\epsilon_{XX<}\epsilon_{FF}$ do we observe PCH surrounding the amphiphilic protein-rich phase, which in turn surrounds the Fibrillarin condensate, consistent with the experimental results in wild type embryos.

##### Parameter range of $\epsilon_{FX}$:

We proceeded to vary the attraction strength between Fibrillarin and amphiphilic protein X within the range of $\epsilon_{FX}=1.5k_{B}T$. When the attraction strength is relatively low $\left(\epsilon_{FX}\leq 0.75k_{B}T\right)$, the fibrillarin-rich phase and the amphiphilic protein-rich phase do not associate with each other. At moderate attraction strengths $\left(\epsilon_{FX}\geq 1k_{B}T\right.$ and $\left(\epsilon_{FX}\leq 1.25k_{B}T\right)$, the Fibrillarin-rich and amphiphilic protein-rich phases partially wet each other. Finally, at higher attraction strengths $\left(\epsilon_{FX}=1.5k_{B}T\right)$, the amphiphilic protein completely wets Fibrillarin (Extended Figure 5E–F).

##### Parameter range of $c_{X}$:

Additionally, we explored variations in the concentration of amphiphilic protein $X\left(c_{X}\right)$. Notably, for higher concentrations of protein $X\left(c_{X}=0.005-0.013\right)$, we observed that heterochromatin tends to completely engulf the amphiphilic protein-rich phase, which in turn engulfs Fibrillarin (Figure 6A).

#### Quantitative Image Analysis

3D measurements were performed using Arivis Vision4D (Zeiss) while ImageJ was used for all 2D measurements. The details of each analysis pipeline used in this study are listed below:

##### Measuring the fraction of the nucleolar edge occupied by HP1a or H3K9me2

###### Figure 1B:

A cluster of nuclei was manually chosen for analyses ~15–30 mins into the specified interphase and defined as “Early Cycle“, while those observed from ~50–70 mins were defined as “Late Cycle“. Preprocessing steps include background subtraction and denoising using Gaussian blur. HP1a and Fibrillarin were segmented in 3D using the “Intensity Threshold Segmenter” with the Auto segmentation method. To calculate the fraction of the nucleolar edge occupied by HP1a, the nucleolus was dilated by 1 pixel and subtracted from the dilated object to create a 1-pixel shell around the nucleolus. The nucleolus shell was intersected with HP1a segments to calculate the volume of the nucleolar shell that overlaps with HP1a.

###### Figure 6D:

Same as above, except the nucleolus was dilated by 2 pixels to create a 2-pixel shell around the nucleolus. The shell was intersected with H3K9me2 segments in nuclei from eye-discs to calculate the fraction of the nucleolar shell that overlaps with H3K9me2.

##### Measuring Distances

###### Extended Figure 1F:

Preprocessing steps include background subtraction and denoising using Gaussian blur. 1.686 or 359bp foci and Fibrillarin were manually segmented using the “Intensity Threshold Segmenter” with the Simple segmentation method. The distance between a Fibrillarin segment and its nearest 1.686 or 359bp locus was measured in 3D using the “Distances” feature in Arivis.

###### Figure 2B:

HP1a and Fibrillarin were segmented using the “Intensity Threshold Segmenter” with the Auto segmentation method. The distance between HP1a and its nearest Fibrillarin segment was measured in 3D using the “Distances” feature in Arivis.

###### Figure 2F:

AAGAG and 1.686 were segmented using the “Intensity Threshold Segmenter” with the Auto segmentation method. The distance between AAGAG and its nearest 1.686 segment was measured in 3D using the “Distances” feature in Arivis.

##### Measuring Aspect Ratio of HP1a

###### Figure 2D:

Individual nuclei were manually selected 15 mins after the start of Cycle 15. Preprocessing steps include background subtraction and denoising using Gaussian blur. HP1a was segmented using Auto thresholding using Otsu. The aspect ratio of the segment was determined using the Analyze Particles feature in Fiji.

##### Line Scans:

###### Figure 3B:

Line scans were generated using the “Plot profile” feature in Fiji. The highest intensity values in the green and magenta channels were each set to 1.0, and the remaining intensities were normalized to it.

##### Pitchoune void formation measurements

###### Figure 5E–G:

To measure the dynamics of the formation of Pitchoune in −rDNA embryos, maximum intensity projections of Amnioserosa were first preprocessed in Fiji using Subtract background and a Gaussian Blur filter. Auto thresholding for each time point was performed using the Yen method. Using Analyze Particles, the area, circularity, and mean intensity of each segment of Pitchoune was extracted. The mean intensity over time was normalized to its value at T=0.

##### Measuring Volume

###### Supplementary Figure 3:

Preprocessing steps include background subtraction and denoising using Gaussian blur. The volume of segments was extracted using Arivis after Fibrillarin was segmented in 3D using the “Intensity Threshold Segmenter” with the Auto segmentation method (Otsu feature).

#### Statistical Analysis

Data were plotted, and statistical analyses were performed using GraphPad Prism8. P-values were calculated using unpaired two-tailed t-tests.

## Extended Data

## Data Availability

All data supporting the results of this study are included in the manuscript. Reagents used in this study are available upon request.
